# Clinical characteristics and treatment outcomes of angioid streak associated choroidal neovascular membrane (AS-CNV): a Zambian case series

**DOI:** 10.11604/pamj.2020.36.294.25065

**Published:** 2020-08-17

**Authors:** Kshitiz Kumar, Santosh Balasubramaniam, Amar Agarwal

**Affiliations:** 1 Disha Eye Hospital, Kolkata, Barrackpore, India,; 2 Dr. Agarwal´s Eye Hospital, Lusaka, Zambia

**Keywords:** Angioid streak, choroidal neovascular membrane, comet-tail lesion, ranibizumab, aflibercept

## Abstract

This case series illustrates clinical features and treatment outcomes of angioid streak associated CNV (AS-CNV) in 3 consecutive patients. Mean age of patients was 43.2 years with one female patient. Bilateral CNV was present in one patient. Comet-tail lesions were present in all cases. No underlying systemic association was found in any of the patients. All patients were treated with 3 loading doses of anti-VEGF injections (ranibizumab in two and aflibercept was used in one case). Subretinal fluid resolved in all cases with no recurrence of CNV activity at mean follow-up of 10.75 months. AS-CNV in Zambian eyes responds favourably to anti-VEGF injections.

## Introduction

Angioid streaks (AS) were first described by Doyne as “irregular radial lines spreading from the optic nerve head to the retinal periphery” and represent visible irregular crack-like dehiscences in Bruch´s membrane that are associated with atrophic degeneration of the overlying retinal pigmented epithelium (RPE) [1-3]. They are often bilateral and are located in the posterior pole. Commonly associated systemic diseases with AS are pseudoxanthoma elasticum (PXE), Paget´s disease of bone and sickle-cell anemia besides others. AS are mostly asymptomatic unless the lesion involves foveola or develop complications like traumatic Bruch´s membrane rupture or macular choroidal neovascular membrane (CNV) [4]. Incidence of CNV in AS is around 42-86% and generally portends a guarded prognosis [5]. A “comet - tail” lesion is a solitary, subretinal, nodular crystalline white body with a tapered white tail of retinal pigment epithelium atrophy extending posterior to the body and are considered pathognomonic of PXE [6,7]. Treatment of AS-CNV remained a challenge earlier, but with the advent of anti-VEGF intravitreal drugs, the long-term efficacy of anti-VEGF therapy to stabilize the CNV and preserve or even improve vision is now proven [8,9]. We present a series of three patients of AS-CNV with comet-tail lesions without pseudoxanthoma elasticum, their clinical characteristics and visual outcome following intravitreal anti-VEGF injections in Zambian setting.

## Methods

Retrospective record collection of consecutive patients with AS-CNV reporting to a tertiary care eye centre in Lusaka, Zambia. Detailed ophthalmic examination at each visit was done. Informed consent was obtained from each patient for any interventional procedure performed as well as for data use.

## Results

Three patients presented with AS-CNV between November 2015 to November 2018. The study adhered to the tenets of declaration of Helsinki. The clinical profiles of three patients are discussed in the following.

**Figure 1:**
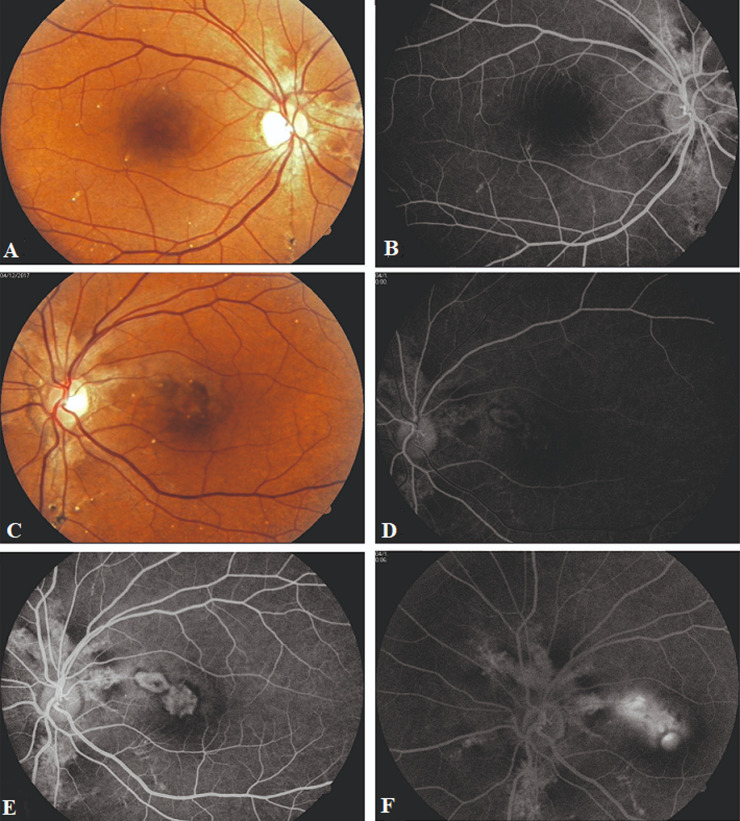
fundus picture and FFA findings of case 1: (A,B) RE posterior pole showing AS with comet-tail lesions, no macular leakage in arteriovenous phase; (C) LE posterior pole fundus pic showing AS with CNV with surrounding retinal haemorrhage; (D,E,F) RE early, mid and late phase FFA showing the extent of CNV with leakage

**Figure 2:**
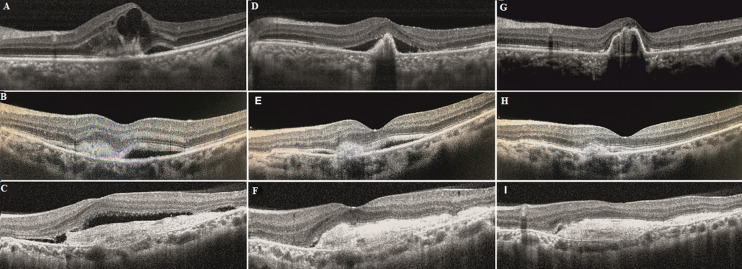
SD-OCT images of three cases; (A,B,C) case 1 showing CME with SRF with type II CNV at presentation, resolving SRF with FVPED at 1 month, resolved SRF with FVPED and foveal thinning at the 3^rd^ month post ranibizumab injections; (D,E,F) case 2 showing small type II CNV with SRF at presentation, decreasing SRF at 1 month and resolved SRF at month 3 post ranibizumab injections; (G,H,I) case 3 showing a large subretinal hyperreflective membrane (SHRM) with SRF at presentation, resolved SRF at 1 month, no SRF and early outer retinal tubule formation at the nasal edge of SHRM at the 3^rd^ month post aflibercept injections

**Case 1:** fourty two year old female presented with blurred vision in the left eye with best corrected visual acuity (BCVA) of 20/60 in left eye (LE) and 20/20 BCVA in right eye (RE). Anterior segment examination was normal. Fundus examination revealed AS radiating from the optic disc in both eyes. LE macula showed CNVM complex with subretinal haemorrhage. Mid-periphery of the retina showed comet-tail lesions in both eyes and more markedly at the posterior pole of RE. Fluorescein angiography showed the linear hyperfluorescent streaks radiating from the optic disc in both eyes. LE macula showed an early hyperfluorescent lesion roughly 1.5DD (disc diameter) with surrounding blocked fluorescence in the early phase, followed by increasing hyperfluorescence in the mid-phase and leakage in the late phase (Figure 1). Spectral-domain optical coherence tomography (SD-OCT) of LE showed fibrovascular pigment epithelial detachment (FVPED) with subretinal fluid and macular edema (central macular thickness (CMT) 342μm) suggestive of type II CNV. Patient underwent 3 consecutive doses of intravitreal anti-VEGF ranibizumab injections at monthly intervals which resulted in drying of the macula with quiescence of the CNVM, CMT 212μm and BCVA improving to 20/30 (Figure 2 A,B,C). No systemic association was there.

**Figure 3:**
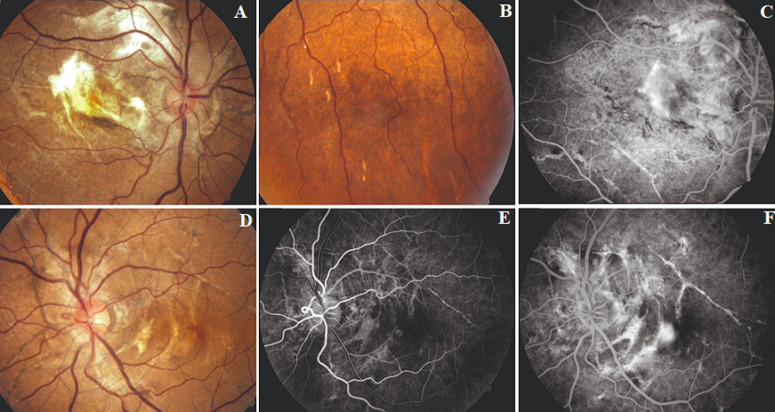
fundus picture and FFA findings of case 2: (A,B,C) RE posterior pole shows AS with disciform scar in macula, multiple comet-tail lesions inferiorly and staining on late phase FFA; (D,E,F) LE posterior pole shows AS with streaks across macula, early and late phase FFA showing staining of the streaks with CNV leakage

**Case 2:** twenty eight year old male patient presented with BCVA 10/200 in RE and 20/20 in LE with recent onset metamorphopsia and micropsia in left eye. There was treatment history of RE CNV with multiple anti-VEGF injections a year before elsewhere. Fundus examination showed AS radiating from the disc in both eyes, disciform scar in the macula in RE, multiple curvilinear angioid streaks in the macula with a small foveal CNV adjacent to 2^nd^ streak in LE and multiple comet-tail lesions in the inferior periphery of both eyes. FFA showed LE early hyperfluoresence with mid phase leak corresponding to CNV and RE staining of the macular scar (Figure 3). SD-OCT in LE showed small FVPED with SRF suggestive of type II CNV with CMT 358μm. Following 3 doses of intravitreal ranibizumab injections at monthly intervals CNV resolved with good recovery of LE BCVA to 20/20 and CMT 220μm (Figure 2 D,E,F). No systemic association was present.

**Figure 4:**
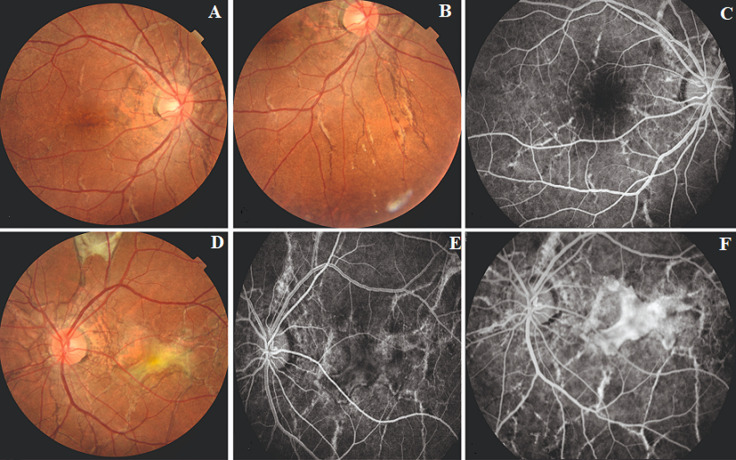
fundus picture and FFA findings of case 3: (A,B) RE posterior pole shows AS, comet-tail lesions in mid peripheral retina; (C) and no macular leakage on FFA; (D,E,F) LE posterior pole shows AS with subretinal hyperreflective membrane in macula, early and mid phase FFA showing leakage from the CNV complex

**Case 3:** thirty eight year old male presented with recent onset diminution of vision in LE with BCVA 20/60 and RE 20/20. Fundus examination showed typical AS in both eyes. LE macula had a large whitish subretinal hyperreflective membrane inter-connected with the angioid streaks, roughly 2DD in size. Comet-tail lesions were present in both eyes inferiorly. FFA showed in LE early hyperfluorescence with mid phase leakage (Figure 4). SD-OCT of LE showed large FVPED with overlying SRF and CMT of 498μm. Post 3 consecutive monthly doses of intravitreal aflibercept injection, SRF resolved, CMT improved to 270μm and BCVA recovered to 20/30 (Figure 2 G,H,I). No systemic association was found.

In all the above three cases, there was no recurrence of AS-CNV activity/new CNV formation in the fellow unaffected eye till mean follow-up period of 10.75±5.03 months (8, 18, 12 months respectively).

## Discussion

This is the first case series from Zambia on treatment naïve AS-CNV describing their outcomes. Clinically none of the patients had any systemic association and in view of pathognomonic comet-tail lesions of PXE, their appearance in isolation without other features of PXE makes an interesting observation. This conforms to the revised criteria for diagnosing PXE where comet lesions constitute only minor eye criteria [10]. Bilateral CNV development was seen in one patient at an interval of 12 months; the available literature predicts risk of bilateralization of CNV within a period of 18-24 months in AS [11]. Anti-VEGF therapy is the treatment of choice as it can stabilize and even improve vision in CNV associated with angioid streaks [12]. In a large series of patients with AS-CNV followed up over 4 years, ranibizumab injections allowed stabilization of BCVA and suppression of angiographic leakage effectively [8]. Recently aflibercept has shown efficacy in treatment of both refractory and treatment naïve AS-CNV [9,13,14]. Aflibercept may offer an additional benefit over other anti-VEGF agents in terms of preventing CNV recurrence as it has a higher affinity for VEGF-A as well as the ability to bind VEGF-B and placental growth factor, resulting in a more effective inhibition of the pathological angiogenic process [9]. Each patient received only 3 loading doses of anti-VEGF therapy (2 patients with ranibizumab and one with aflibercept) without further recurrences of AS-CNV till the last follow-up which was encouraging knowing the fact that CNV in AS has a high tendency of reactivation/recrudescence [8,12]. The fewer number of injections required over a mean period of 10 months points towards the nature of AS-CNV to be closer to myopia-related CNV than nAMD-related CNV [8].

## Conclusion

This series highlights the association comet-tail lesions in AS-CNV in the absence of clinical PXE, with favourable response of CNV in angioid streak to anti-VEGF ranibizumab/aflibercept with no recurrence. However, further longitudinal studies with a larger cohort and extended follow-up are required to know the long term behaviour of AS-CNV in Zambian eyes.

### What is known about this topic

- Choroidal neovascularization (CNV) complicating AS (prevalence: 42%-86%) is an important cause of legal blindness and financial burden in middle-aged individuals affected by this condition;
- “Comet-tail” lesions are considered pathognomonic of PXE;
- The efficacy of anti-vascular endothelial growth factor (VEGF) agents, such as bevacizumab or ranibizumab, in the treatment of AS-associated CNV has been evaluated, with ranibizumab recently receiving official approval for this condition but there is still a high tendency of reactivation or recrudescence.

### What this study adds

- This is the first series of AS-CNV from Zambia describing clinical profile and treatment outcomes; also it´s a well documented series of 4 eyes in African patients which to date is the largest number described;
- This series highlights presence of “Comet-tail” lesions in absence of PXE in African population;
- This case series shows good response of Anti-VEGF agents ranibizumab and aflibercept in AS-CNV with no incidence of reactivation; it establishes the potential role of aflibercept in AS-CNV which has been described in very limited studies so far; thus favourable outcomes can be expected in African eyes with AS-CNV on treatment with anti-VEGF agents.
